# miR-199a-5p Represses Protective Autophagy and Overcomes Chemoresistance by Directly Targeting DRAM1 in Acute Myeloid Leukemia

**DOI:** 10.1155/2019/5613417

**Published:** 2019-09-15

**Authors:** Yang Li, Guojun Zhang, Bin Wu, Wei Yang, Zhuogang Liu

**Affiliations:** Department of Hematology, Shengjing Hospital of China Medical University, Shenyang 110004, China

## Abstract

Chemotherapy resistance is still a primary clinical obstacle to the successful treatment of acute myeloid leukemia (AML). The underlying mechanisms of drug resistance are complicated and have not been fully understood. Here, we found that miR-199a-5p levels were significantly reduced in refractory/relapsed AML patients compared to those who achieved complete remission after chemotherapy. Consistently, miR-199a-5p was markedly decreased in Adriamycin-resistant AML K562/ADM cells in contrast with Adriamycin-sensitive K562 cells, and its decrement dramatically correlated with the chemoresistance of AML cells. Furthermore, we demonstrated that the basic and Adriamycin-induced autophagic activity in K562/ADM cells was higher than that in K562 cells. This inducible autophagy played a prosurvival role and contributed to the development of acquired drug resistance. Importantly, we investigated that miR-199a-5p could negatively regulate autophagy, at least in part, by inhibiting damage regulator autophagy modulator (DRAM1) expression at both the transcriptional and posttranscriptional level. miR-199a-5p bound directly to the 3′-UTR of DRAM1 mRNA which was a functional target of miR-199a-5p. Indeed, downregulation of DRAM1 gene by siRNA in K562/ADM cells resulted in autophagy suppression and chemosensitivity restoration. These results revealed that the miR-199a-5p/DRAM1/autophagy signaling represented a novel pathway regulating chemoresistance, indicating a potential therapeutic strategy for the intervention in drug-resistant AML.

## 1. Introduction

Acute myeloid leukemia (AML) is an aggressive hematological malignancy characterized by differentiation arrest and unlimited proliferation of clonal myeloid precursors. It manifests a remarkable heterogeneity with great variability in clinical course and response to therapy. Despite great advances recently reported in treatment regimens, systemic chemotherapy still remains as the standard initial management for newly diagnosed AML patients. Unfortunately, some patients will suffer from disease relapse subsequently even though they had a good response to initial treatment. This is partially due to the development of acquired chemoresistance induced by the administration of repetitive cycles of chemotherapeutic agents. Such acquired chemoresistance is one of the major obstacles in the successful treatment of AML. However, the exact mechanisms underlying chemoresistance are complicated and have not been completely assessed, confirming the urgent need to identify the associated cellular and molecular mechanisms to explore new strategies that are able to interfere with that process.

Recent and numerous evidences have indicated that microRNAs (miRNAs) exert important functions in regulating tumor cell sensitivity to drugs in various human cancers including AML [1, 2]. miRNAs are an evolutionarily conserved group of short endogenous noncoding RNAs of 18–25 nucleotides length which are known to mediate the expression of target transcripts at the transcriptional or posttranscriptional level by promoting their degradation or suppressing their translation [3]. Previous studies demonstrated that aberrant miRNA expressions were involved in cancer initiation, development, progression, and especially drug resistance [4]. For example, miR-181a, which is overexpressed in T-cell leukemia/lymphoma, functions as a critical regulator of chemosensitivity through modulation of the AKT pathway [5]. miRNA let-7a is found to be regulated by SDF-1*α*/CXCR4 signaling in human AML cells, and overexpression of let-7a is associated with decreased chemoresistance via promoting cytarabine-induced apoptosis both in vitro and in vivo [6]. Among these miRNAs, miR-199a-5p is of particular interest because it is downregulated in many types of human cancers including osteosarcoma, human hepatocellular carcinoma, and ovarian cancer [7–11]. Recently, an investigation analyzed miRNA expression difference between newly diagnosed AML patients and healthy donors using the quantitative reverse transcription polymerase chain reaction (RT-PCR) for 365 human miRNAs. This study identified miR-199a-5p as one of the subsets of miRNAs which were significantly downregulated in AML patients [12]. Moreover, data from miRNA microarray analysis showed that miR-199a-5p was one of the downregulated miRNAs in normal karyotype AML patients compared with abnormal karyotype patients, indicating that miR-199a-5p could be a potential tumor suppressor in AML and has a critical role in leukemogenesis dependent of cytogenetics [13]. Notably, the aberrant miR-199a-5p expression has been linked to chemotherapy resistance in a variety of cancers [9, 14, 15]. One relevant explanation for that is miR-199a-5p plays a crucial role on the autophagy level by directly targeting multiple different genes in the autophagic signaling pathway [9, 14, 16, 17]. Growing evidence indicated that high level of autophagy induced by cytotoxic drugs was considered to be a prosurvival mechanism in tumor cells [18, 19]. Therefore, autophagy inhibition might have the potential to improve the chemotherapy effect [20, 21]. Despite all these studies, the detailed mechanism of miR-199a-5p, as well as the role of autophagy in AML chemoresistance regulation, is largely unknown and needs to be discovered.

The present study was undertaken to determine miR-199a-5p expression and autophagy level separately in human leukemia cells, sensitive to Adriamycin cell lines (K562) and their Adriamycin-resistant counterpart (K562/ADM) cells, and to define the roles of miR-199a-5p and autophagy in chemoresistance of AML cells and their correlation and associated signaling pathway. Here, we have demonstrated that downregulated miR-199a-5p expression and activated autophagy were significantly associated with acquired chemoresistance in AML cells. Furthermore, miR-199a-5p expression adversely modulated autophagy by targeting the DRAM1 gene directly, providing a valuable strategy for the treatment of drug-resistant AML patients.

## 2. Materials and Methods

### 2.1. Clinical Samples

Bone marrow samples were collected from 32 relapsed/refractory AML patients and 11 complete remission AML patients who were hospitalized in the Affiliated Shengjing Hospital of China Medical University from June 2015 to June 2016. The acute promyelocytic leukemia (M3) was excluded because of different pathogenesis. Relapsed AML and refractory AML meet the diagnostic criteria as previously reported [22]. This study was approved by the Institute's review board of China Medical University and conducted in accordance with the Helsinki Declaration. The informed consent was obtained from all patients. The characteristics of all AML patients are summarized in Table 1.

### 2.2. Cell Culture and Treatments

The acute human erythroid leukemia cell line K562 and its Adriamycin-resistant counterpart K562/ADM were kindly gifted by Prof. Yanqiu Zhang (Institute of Hematology, Chinese Academy of Medical Sciences, Tianjin). Cells were cultured in the complete RPMI-1640 medium supplemented with 10% fetal bovine serum (FBS), 100 U/ml penicillin, 100 *μ*g/ml streptomycin, and 2 mM L-glutamine at 37°C in a 5% CO_2_ humidified atmosphere and were routinely subcultured every 2-3 days. K562/ADM cells were routinely maintained in the medium containing 5 *μ*g/ml Adriamycin (ADM) to ensure the drug-resistant phenotype and incubated in drug-free medium for 2 weeks prior to experiments. Cells harvested during their logarithmic growth phase were used in experiments.

### 2.3. Drug Sensitivity Assay

The cytotoxicity of ADM was evaluated by the methyl thiazolyl tetrazolium (MTT) assays in K562 and K562/ADM cells. Cells were seeded into 96-well plates (5,000 cells/well) and incubated for 24 h at 37°C and 5% CO_2_. The cells were then exposed to graded concentrations of ADM at 0.01 *μ*g/ml, 0.1 *μ*g/ml, 1 *μ*g/ml, 5 *μ*g/ml, 10 *μ*g/ml, 50 *μ*g/ml, and 100 *μ*g/ml for 24 hours (3 replicates for each concentration). 10 *μ*l of MTT solution (5 mg/ml) was added to each well, and the plates were incubated for an additional 4 hours at 37°C and 5% CO_2_. 100 *μ*l 10% sodium dimethyl sulfoxide (DMSO) was added to fully dissolve the formazan pellet. The absorbance was measured at a wavelength of 490 nm by a microplate reader three times, and the average values were used to construct the growth inhibition curve of cells. Cell inhibitory ratio (%) = (A570_sample_ − A570_blank_)/(A570_control_ − A570_blank_) × 100%. The half inhibitory concentration (IC_50_) of ADM was calculated as previously described [23].

### 2.4. Cell Transfection

For the miR-199a-5p and DRAM1 functional analysis, cells were transfected with miR-199a-5p mimic, miR-199a-5p inhibitor, nontargeting control miRNA mimic/inhibitor, and 3 short interfering RNA (siRNA) against DRAM1 gene (Sangon Biological Engineering Technology, Shanghai, China) using lipofectamine 2000 (Invitrogen) according to the manufacturer's instructions.

### 2.5. Quantitative Real-Time RT-PCR Assay

Total RNAs from cells were extracted using the TRIzol reagent (Invitrogen) according to the manufacturer's protocol. The integrity and quantity of extracted RNA were assessed on a Nano Drop 1000 spectrophotometer (Thermo Fisher Scientific, Inc.) Reverse transcription reactions were performed with the First Strand cDNA Synthesis Kit (Amersham Biosciences). The relative quantity of miR-199a-5p was detected with Power Taq PCR Master Mix (Applied Biosystems) on ABI PRISM 7500 Sequence Detection System (Applied Biosystems) following the manufacturer's instructions. DRAM1 mRNA was measured by qRT-PCR using the Power QuantiTechTMSYBR Green PCR Kit (Applied Biosystems). The PCR primers were designed and synthetised by Sangon Biological Engineering Technology (Shanghai, China). U6 and *β*-actin were used as endogenous reference genes to normalize miRNA and mRNA expression level, respectively. The relative expression of miR-199a-5p or DRAM1 mRNA was analyzed using the cycle threshold method [24]. All reactions were performed in triplicates. Primer and probe sequences are shown in Table 2.

### 2.6. Western Blot Analysis

Cell were washed in cold PBS buffer for 3 times and lysed in ice-cold RIPA buffer on ice for 30 min. Cell debris was discarded by centrifugation at 12,000 rpm for 10 min at 4°C. The protein concentration was determined using the BCA protein quantitation kit. Protein samples were electrophoresed on SDS-PAGE and transferred onto PVDF membranes. Immunoblotting was performed according to the manufacturer's instructions. The primary antibodies were LC3II, LC3I, BECN1, P62, and DRAM1 (Abcam). The anti-*β*-actin (Santa Cruz) was used to normalize the amount of the analyzed samples.

### 2.7. mRFP-GFP-LC3 Assay

The K562 and K562/ADM cells were plated on the cover slip of the 6-well plate and allowed to reach 50%–70% confluence at the time of transfection. mRFP-GFP-LC3 adenoviral vectors were obtained from HanBio Technology Co. Ltd. (HanBio, Shanghai, China). Adenoviral infection was performed following the manufacturer's instructions. Cells were incubated in the growth medium with mRFP-GFP-LC3 adenovirus at 30 multiplicities of infection (MOI) for 4 h and were then grew in a new medium for another 24 h. After infection, cells were treated with different reagents according to different groups. Autophagy was observed under a fluorescence microscope (Olympus). Autophagic flux was then measured by evaluating the number of GFP, RFP, and merged points (point/cell were counted) [25].

### 2.8. Dual Luciferase Reporter Assay

The 3′-UTR of DRAM1 containing the miR-199a-5p binding sites and its corresponding mutated sequence were cloned into the pmirGLO luciferase reporter vector (Promega) downstream of Renilla luciferase named DRAM1-3′-UTR and DRAM1-3′-UTR mut, respectively. Using lipofectamine 2000 (Invitrogen), K562/ADM cells were cotransfected with the reporter constructs and miR-199a-5p mimics, nontargeting control (NC), or the mimic NC. Luciferase activity was determined after 48 h using the Dual-Luciferase Reporter Assay System (Promega) according to the manufacturer's protocol. Data are presented as the ratio of experimental (Renilla) luciferase to control (Firefly) luciferase.

### 2.9. Assessment of Cell Viability with a CCK-8 Assay

Cell viability assays were performed by using Cell Counting Kit-8 (Sigma) in accordance with the manufacturer's instructions. Cells were seeded in 96-well plates at a density of 5 × 10^4^/ml. After treatment, the viable cells were counted by absorbance measurements at a wavelength of 450 nm at the indicated time points. The optical density value was reported as the percentage of cell viability in relation to the control group.

### 2.10. Statistical Analysis

All statistical analyses were performed using the SPSS software (version 19.0). Data were expressed as the means ± standard deviation from at least 3 separate experiments. Differences were analyzed by Students' *t*-test between two groups or by one-way analysis of variance (ANOVA) among multiple groups. *P* values <0.05 are considered significant.

## 3. Results

### 3.1. miR-199a-5p Is Downregulated in AML Clinical Samples and Cell Lines

**Figure 1:**
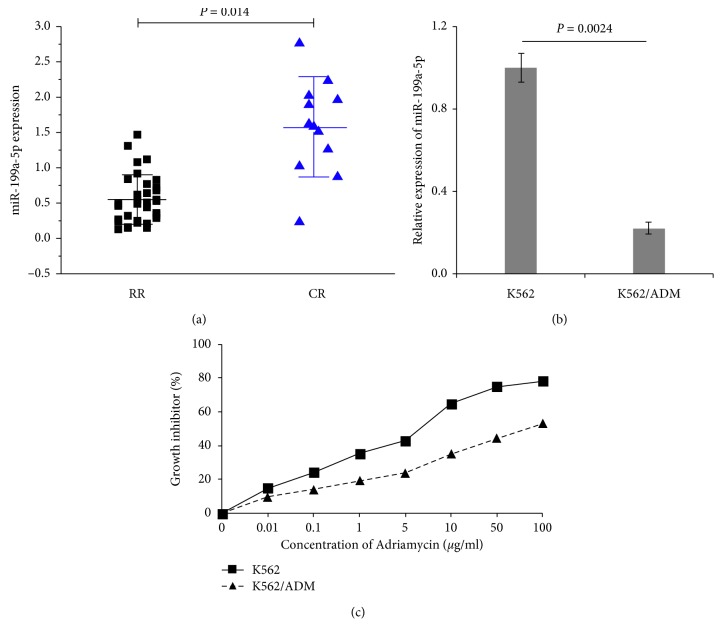
Relative expression of miR-199a-5p in leukemia cell lines and AML samples. miR-199a-5p was measured by qRT-PCR in 32 relapsed/refractory AML bone marrow samples and 11 complete remission AML bone marrow samples (a) and K562 and K562/ADM cells (b) using U6 for normalization. (c) The inhibitory effect of ADM on K562 and K562/ADM cells was detected by the MTT assay. After treatment with various concentrations of ADM, growth inhibition was assessed. IC_50_ of K562/ADM (146.14 ± 0.079 *μ*g/ml) was 48.7-fold than that of K562 (3.08 ± 0.056 *μ*g/ml).

To determine whether miR-199a-5p was involved in regulating the sensitivity of leukemia cells to chemotherapeutic regimens, we initially evaluated the expression levels of miR-199a-5p in bone marrow samples isolated from 32 relapsed/refractory (RR) and 11 complete remission (CR) AML patients by qRT-PCR. The characteristics of the study subjects are summarized in Table 2. As shown in Figure 1, we found that the expression of miR-199a-5p was distinctly decreased in RR patients compared with that in CR patients (mean ± SD: 0.548 ± 0.350 vs 1.579 ± 0.707, *P* < 0.05). To further confirm the correlation, the expression of miR-199a-5p in drug-sensitive and drug-resistant leukemia cell lines was examined as well. We first compared the ADM sensitivity between ADM-sensitive and -resistant leukemia cells by measuring the cells growth inhibition after different doses of ADM treatment for 48 h (Figure 1(c)). It suggested that the drug sensitivity of K562/ADM was significantly lower than that of K562 cells, indicating K562/ADM cells have more potential for resistance to ADM than K562 cells. We identified a more significant difference between the pair of leukemia cells than that of the clinical samples. miR-199a-5p expression in K562/ADM cells was strikingly downregulated, only about one-fifth of K562 cells (Figure 1(b)). These results suggested that the reduced miR-199a-5p might contribute to the development of chemoresistance in AML.

### 3.2. Overexpression or Silencing of miR-199a-5p Affects AML Cell Sensitivity to ADM

**Figure 2:**
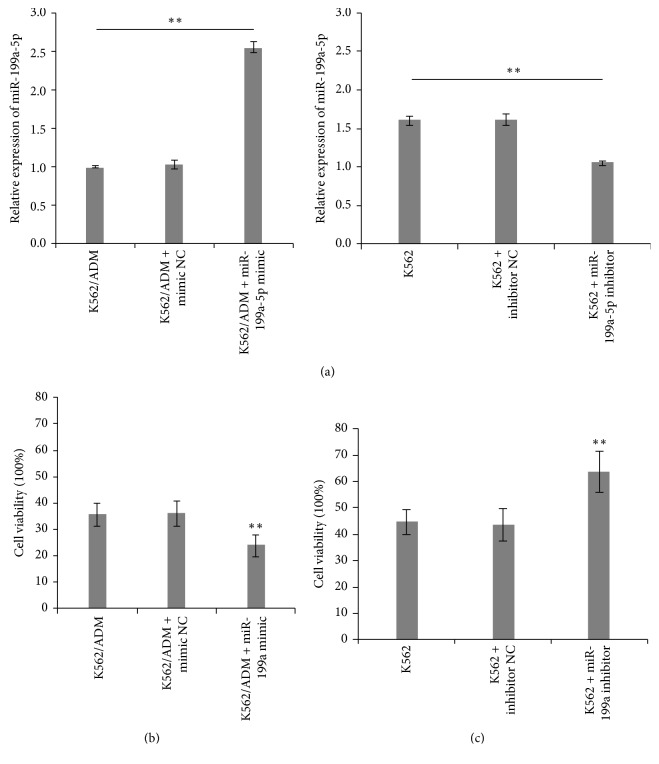
(a) qRT-PCR analysis of transfection efficiency of miR-199a-5p mimic in K562/ADM cells and miR-199a-5p inhibitor in K562 cells. (*n* = 3; ^*∗∗*^*P* < 0.05). (b) K562/ADM cells were transfected with miR-199a-5p mimic or negative control (NC) mimic and subsequently treated with 140 *μ*g/ml of ADM for 48 h, and cells viability was evaluated by the CCK-8 assay (*n* = 3; ^*∗∗*^*P* < 0.05). (c) K562 cells were transfected with miR-199a-5p inhibitor or NC inhibitor with 1.5 *μ*g/ml of ADM for 48 h, and cells viability was evaluated by the CCK-8 assay (*n* = 3; ^*∗∗*^*P* < 0.05).

To confirm the involvement of miR-199a-5p in regulating ADM sensitivity in AML cells, we overexpressed or silenced its expression in K562/ADM and K562 cells, respectively. Then, cells were transfected with miR-199a-5p mimic, inhibitor, or the corresponding negative control. The qRT-PCR result shows that the induction of miR-199a-5p mimic significantly increased miR-199a-5p expression in K562/ADM cells while miR-199a-5p inhibitor reduces miR-199a-5p expression in K562 cells (Figure 2(a)). The subsequent CCK-8 cell proliferation assay revealed that enforced expression of miR-199a-5p markedly increased the sensitivity of K562/ADM cells to ADM treatment compared with their vector controls. Conversely, the silencing of miR-199a-5p expression in K562 cells was able to decrease the drug sensitivity (Figures 2(b) and 2(c)). The above evidence indicates that upregulation of miR-199a-5p leads to reduced chemoresistance while downregulation of miR-199a-5p results in enhanced chemoresistance upon ADM treatment, showing a significant association of miR-199a-5p expression with ADM chemoresistance in AML cells.

### 3.3. Autophagy Is Induced by ADM and Inhibition of Autophagy Partially Reverse Chemotherapy Resistance in AML Cells

**Figure 3:**
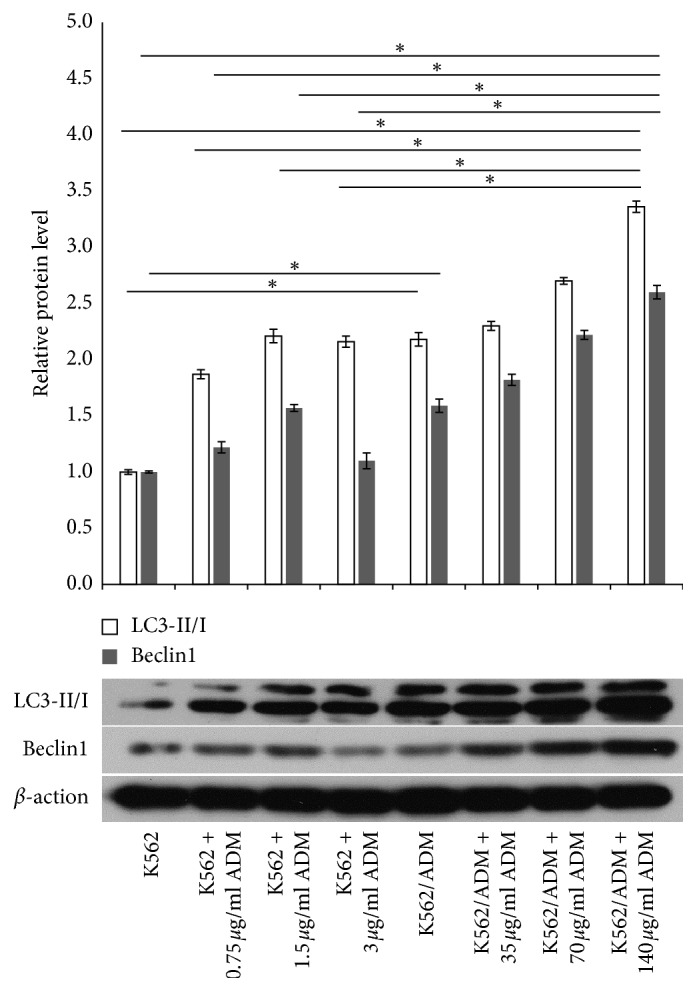
Western blot analysis of LC3-II/I and Beclin1 protein was performed in K562 and K562/ADM cells at the baseline and after ADM treatment in different concentrations for 48 h. Nontreated K562 cells were used as control (*n* = 3; ^*∗*^*P* < 0.05).

**Figure 4:**
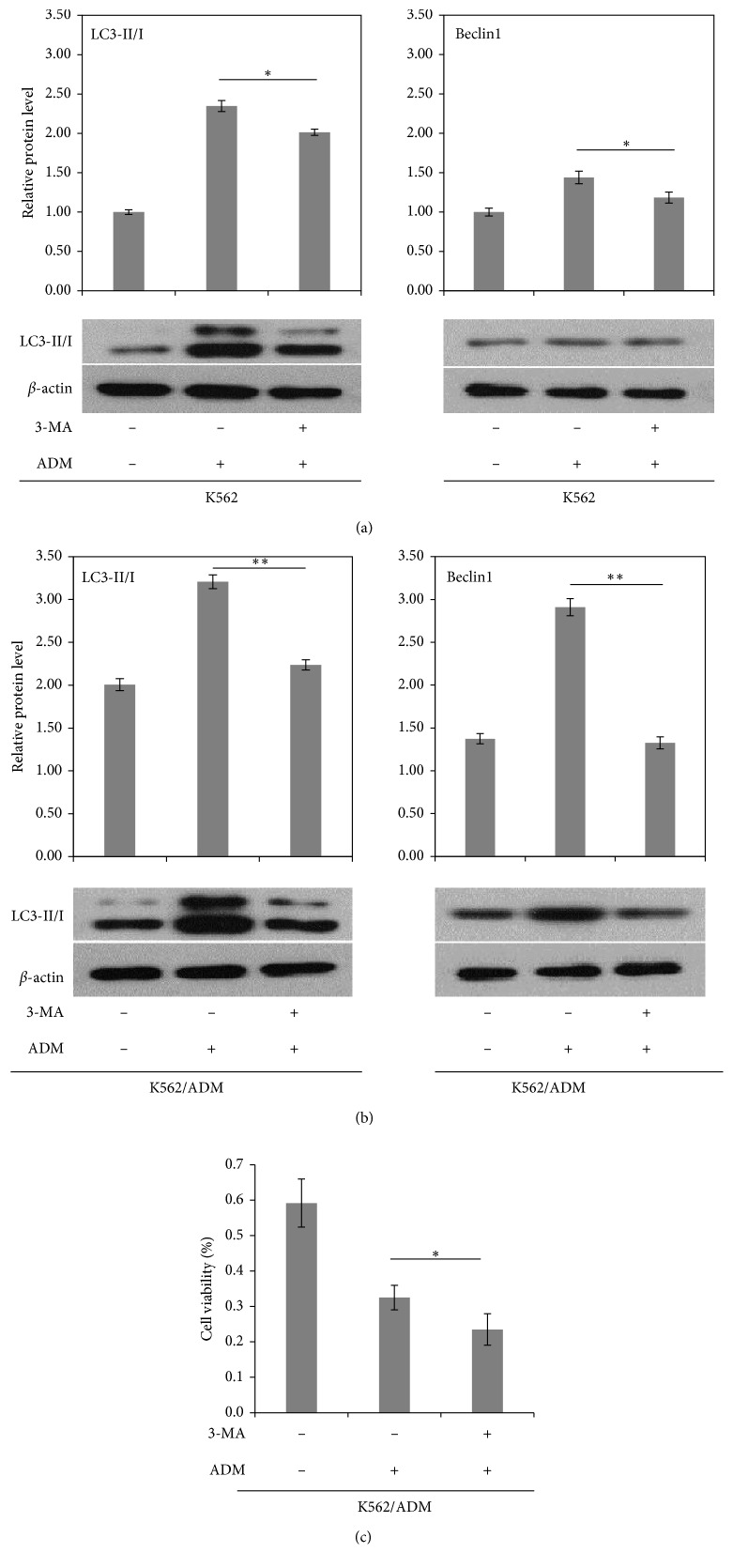
Western blot assay of LC3-II/I and Beclin1 protein under treatment of 3-MA was performed in K562 (a) and K562/ADM cells (b) (*n* = 3, ^*∗*^*P* < 0.05, ^*∗*^*P* < 0.01). (c) K562/ADM cell viability was examined using a CCK-8 assay under the combined treatment of ADM (140 *μ*g/ml) and 3-MA. Nontreated K562/ADM cells were used as control (*n* = 3; ^*∗*^*P* < 0.05).

Accumulating evidence indicates that treatment with cytotoxic agents in cancer cells induces autophagy. However, different anticancer drugs induce different effects of autophagy in different cancer types, either prodeath or prosurvival [26, 27]. To explore the relationship between autophagy and ADM resistance in AML cells, western blot analysis was performed to determine the expression of Beclin1 and LC3II/I under the basic state or after different concentrations of ADM treatment. Autophagy initiation relies on two important processes: phagophore formation and elongation. The formation of a phagophore is promoted by activation of Vps34-Beclin1-P150 complex, and the phagophore elongation is mediated by its PE-conjugated LC3-II protein system. LC3-I is conjugated to phosphatidylethanolamine (PE) and recruited to the autophagosomes to form membrane-bound LC3-II. Both Beclin1 expression and LC3II/I ratio are positively related with autophagy which are widely used to monitor autophagic activity as critical biological markers [28]. As indicated in Figure 3, K562/ADM cells showed a higher ratio of LC3-II to LC3-I and a higher expression of Beclin1 when compared with K562 cells at the baseline. We further discovered that ADM treatment with different doses generally induced a promotion in the LC3-II/I ratio and Beclin1 expression in both cell lines with the exception of 3 *μ*g/ml ADM in K562 cells. This is probably because the relatively high concentration of ADM for K562 cells which may induce cell necrosis or apoptosis, not autophagy. Particularly, K562/ADM cells treated with 140 *μ*/ml ADM presented significantly higher autophagic activity than all ADM concentration groups of K562 cells which may explain why K562/ADM cells possess potent resistance to extra stressors. Subsequently, we pretreated the cells with 3-methyl adenine (3-MA) for 3 hours before exposure to ADM, and we observed that both of LC3-II/LC3-I ratio and Beclin1 level were significantly suppressed in K562/ADM and K562 cells (Figures 4(a) and 4(b)), reconfirming that ADM induces autophagy in AML cells which can be prevented by the autophagy inhibitor. Furthermore, we investigated the effects of ADM on K562/ADM cell viability when autophagy is inhibited using a CCK-8 assay. We found that ADM decreased K562/ADM cell survival, while these effects were augmented when autophagy was suppressed by 3-MA (Figure 4(c)). These results revealed that autophagy acted as a protective means of promoting cell survival in response to chemotherapy in chemoresistant leukemia cells, indicating autophagy inhibition may represent an important therapeutic strategy to overcome chemotherapy resistance.

### 3.4. miR-199a-5p Regulates Autophagy in AML Cells

**Figure 5:**
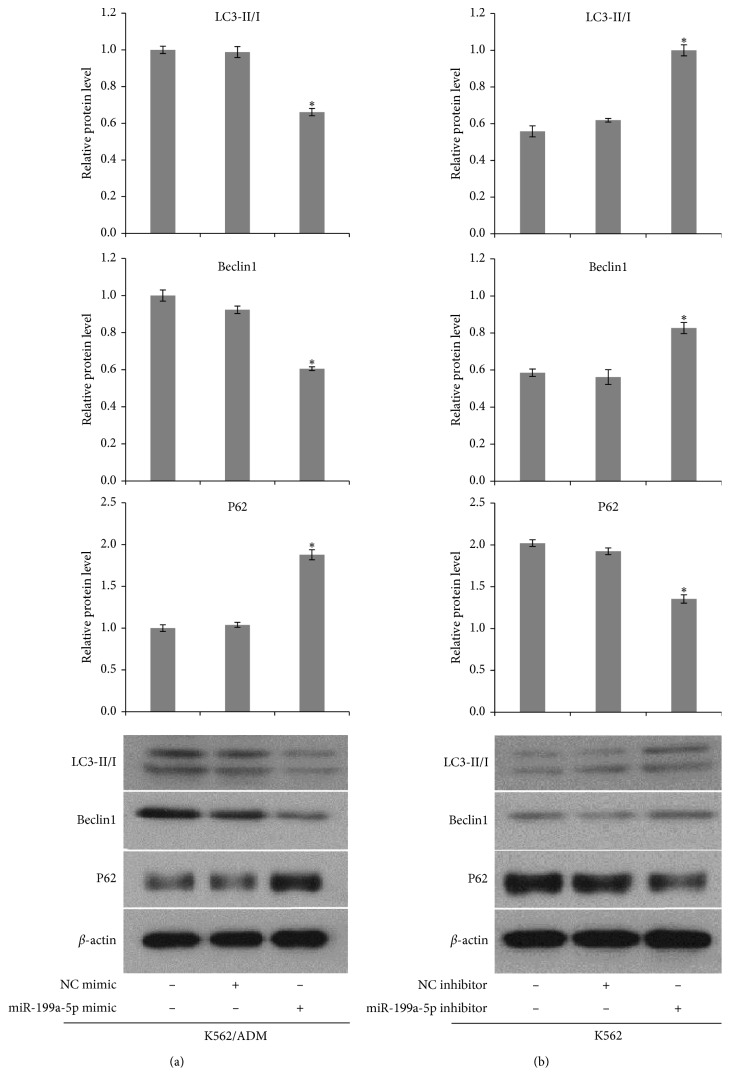
Western blot assay was conducted to examine the protein levels of LC3II/I ratio, Beclin1, and P62 in K562 and K562/ADM cells after they were transfected with the miR-199a-5p inhibitor, mimic, or miR-NC for 24 h. Data were normalized to *β*-actin. The untreated cell group was set as the control group (*n* = 3; ^*∗*^*P* < 0.05).

**Figure 6:**
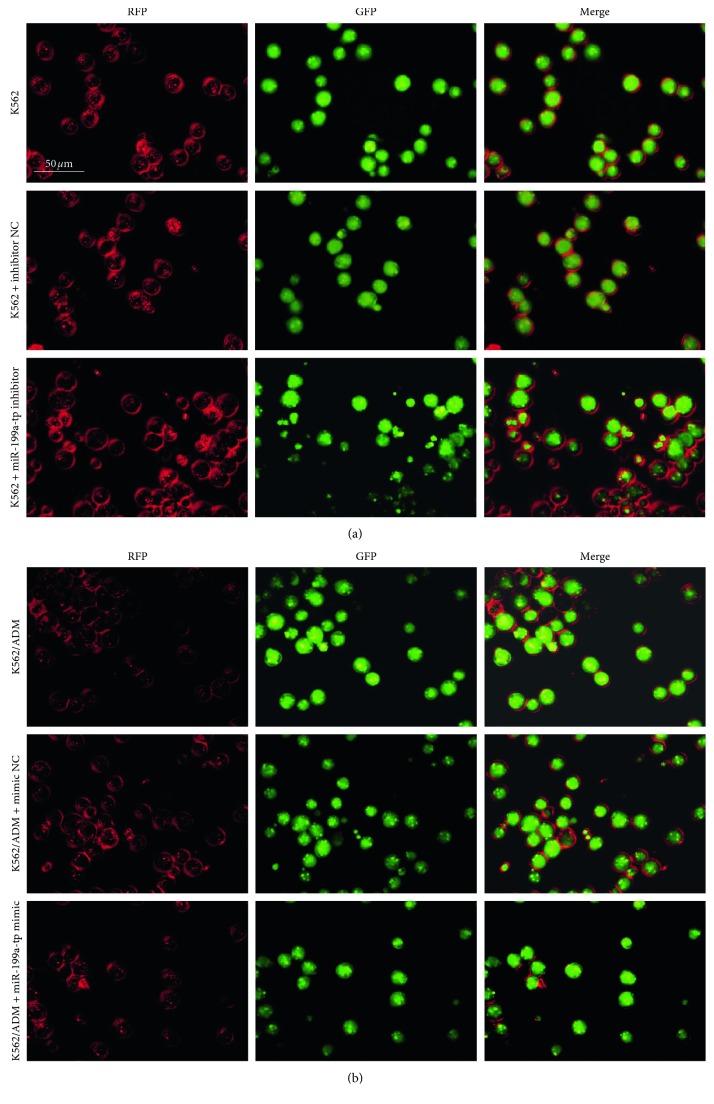
After transfected with miR-199a-5p mimic and inhibitor in K562/ADM and K562 cells, respectively, the cells were subsequently infected with mRFP-GFP-LC3 adenovirus and were observed under a confocal fluorescence microscope. Representative images are presented to indicate the cellular localization patterns of mRFP-GFP-LC3 fusion protein.

To investigate whether miR-199a-5p is involved in autophagy regulation in leukemia cells, we detected the protein levels of LC3II/LC3I ratio, Beclin1, and P62 which are recognised as classical autophagy-related markers by the western blot assay. K562 and K562/ADM cells were firstly transfected with miR-199a-5p inhibitor, miR-199a-5p mimic separately, or the corresponding miR control (NC-mimic/NC-inhibitor). Both the miR-199a-5p mimic and inhibitor were successfully transfected in the two cell lines (Figure 2(a)). Western blot results demonstrated that, compared with the control group, transfection with miR-199a-5p mimic in K562/ADM cells led to a significant downregulation in the protein levels of LC3II/LC3I and Beclin1 and upregulation in P62 expression (Figure 5). Conversely, inhibition of miR-199a-5p shows the opposite effect. Transfection with the miR-199a-5p inhibitor in K562 cells induced an approximately 1.8-fold increase in the level of LC3II/I and a 1.4-fold increase in the level of Beclin1, whereas P62 was significantly reduced (*P* < 0.05) (Figure 5). The above findings indicated that miR-199a-5p might be a negative regulator of autophagy. To further confirm the effect of miR-199a-5p inhibition on autophagy, mRFP-GFP-LC3 adenovirus was used to infect K562 and K562/ADM cells after they were, respectively, transfected with miR-199a-5p inhibitor and miR-199a-5p mimic. GFP is sensitive to the acidic pH inside the lysosome and will lose fluoresce once an autophagosome fuses with the lysosome to form an autolysosome. However, RFP is more stable and resistant to the low PH, and autolysosomes stain as red only. In green/red merged images, yellow puncta (RFP+/GFP+) indicate autophagosomes, while red puncta (RFP+/GFP−) indicate autolysosomes. So, if autophagy is induced or inhibited from initiation, both of yellow and red puncta will increase or decrease correspondingly. However, if autophagy is blocked by autophagosome degradation, the yellow puncta are going to increase while the red puncta remain constant or decrease [29]. We finally analyzed autophagic flux in the two cell lines according to the fluorescence images. As shown in Figure 6, compared with their respective controls, the levels of autophagosomes (yellow dots in merged images) and autolysosomes (red dots in merged images) were notably increased in K562 cells after transfection with the miR-199a-5p inhibitor. In contrast, K562/ADM cells transfected with miR-199a-5p mimic exhibited significant attenuation of both autophagosomes and autolysosomes. All together, these results suggest that enforced expression of miR-199a-5p inhibits autophagy, which is precisely the result of preventing autophagy initiation instead of blocking autophagosome degradation.

### 3.5. miR-199a-5p Suppressed DRAM1 Expression by Directly Targeting DRAM1 3′UTR

**Figure 7:**
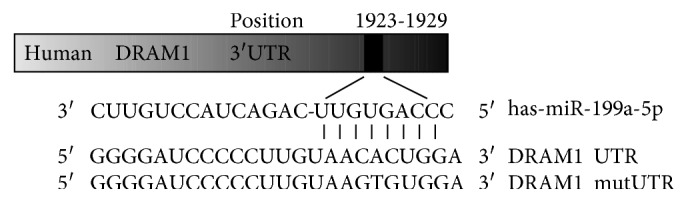
Sequence indicating the wild-type and mutated-type binding sites of DRAM1 3′UTR in miR-199a-5p.

**Figure 8:**
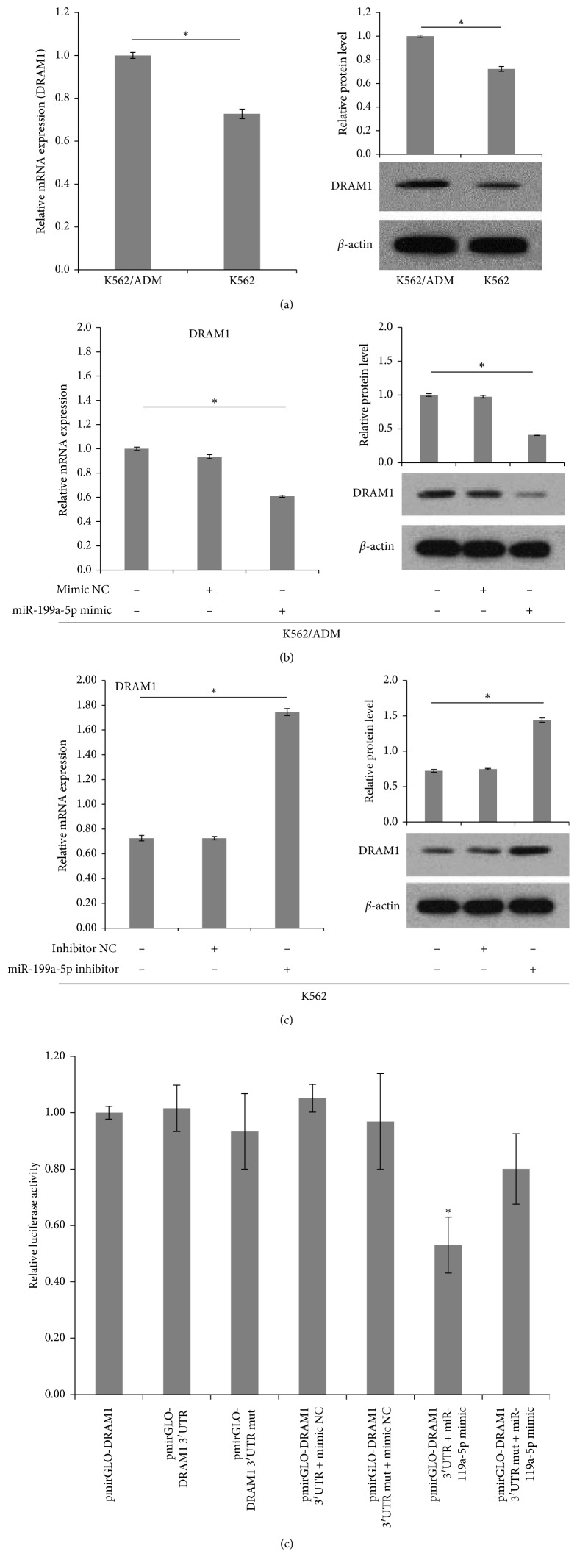
(a) RT-PCR and western blot analysis were conducted to examine basic DRAM1 expression in K562 and K562/ADM cells (*n* = 3; ^*∗*^*P* < 0.05). (b) RT-PCR and western blot analysis were performed to determine DRAM1 expression when K562/ADM cells were transfected with miR-199a-5p mimic or mimic NC (*n* = 3; ^*∗*^*P* < 0.05). (c) RT-PCR and western blot analysis were performed to determine DRAM1 expression when K562 cells were transfected with miR-199a-5p inhibitor or inhibitor NC (*n* = 3; ^*∗*^*P* < 0.05). (d) Dual luciferase reporter assay of K562/ADM cells cotransfected firefly luciferase construct containing DRAM1 3′-UTR or mutant DRAM1 3′-UTR along with miR-199a-5p mimic or mimic NC. Normalized luciferase activity of empty control vector- (pmirGLO-) transfected cells is set to 1.0 (*n* = 3; ^*∗*^*P* < 0.05).

To elucidate the molecular mechanism by which miR-199a-5p functioned as a negative regulator in autophagy, bioinformatic databases such as TargetScan, Pictar, and miRBase were searched to predict the potential targets of miR-199a-5p. Among the list of putative targets, DRAM1 was predicted to be a target gene of miR-199a-5p. DRAM1 contains a highly conserved miR-199a-5p binding site at nucleotides 1923–1929 in its 3′UTR (Figure 7) and has been reported to be involved in cell autophagy [30, 31]. A further hint for the role of DRAM1 gene as a potential target of miR-199a-5p lies in the PCR and western blot analysis that reveals both mRNA and protein expression of DRAM1 are downregulated in K562 cells compared with their drug-resistant counterpart K562/ADM cells (Figure 8(a)), showing an inverse association with miR-199a-5p expression. To further confirm the influence of miR-199a-5p in regulating DRAM1 expression, we performed a functional analysis by either overexpressing or inhibiting miR-199a-5p. Transfection of the miR-199a-5p inhibitor in K562 cells led to a significant increase in DRAM1 expression, at both mRNA and protein level. Conversely, enforced expression of miR-199a-5p in K562/ADM cells efficiently inhibited DRAM1 mRNA and protein expression (Figures 8(b) and 8(c)). Although miRNAs regulate gene expression mostly at the translational level, it is becoming clear that miRNAs can also negatively regulate gene expression through effects on mRNA degradation [32–35]. Our findings suggested that miR-199a-5p influences DRAM1 mRNA degradation in addition to posttranscriptional level inhibition. To prove a direct interaction between miR-199a-5p and its binding site within DRAM1 mRNA, we performed the luciferase reporter assay using K562/ADM cells. As exhibited in Figure 8(d), cotransfection with miR-199a-5p mimic and wild-type DRAM1 vector significantly reduced the luciferase activity by greater than 40% compared to the vector control. However, there was no significant difference in luciferase activity when cells were cotransfected with the mutated DRAM1 3′-UTR and miR-199a-5p. Taken together, these findings implied that miR-199a-5p might attenuate the expression of DRAM1 by directly targeting the DRAM1 3′-UTR.

### 3.6. DRAM1 Silencing Reduced the Autophagy Activity and Improved the Resistance of K562/ADM Cells to ADM

**Figure 9:**
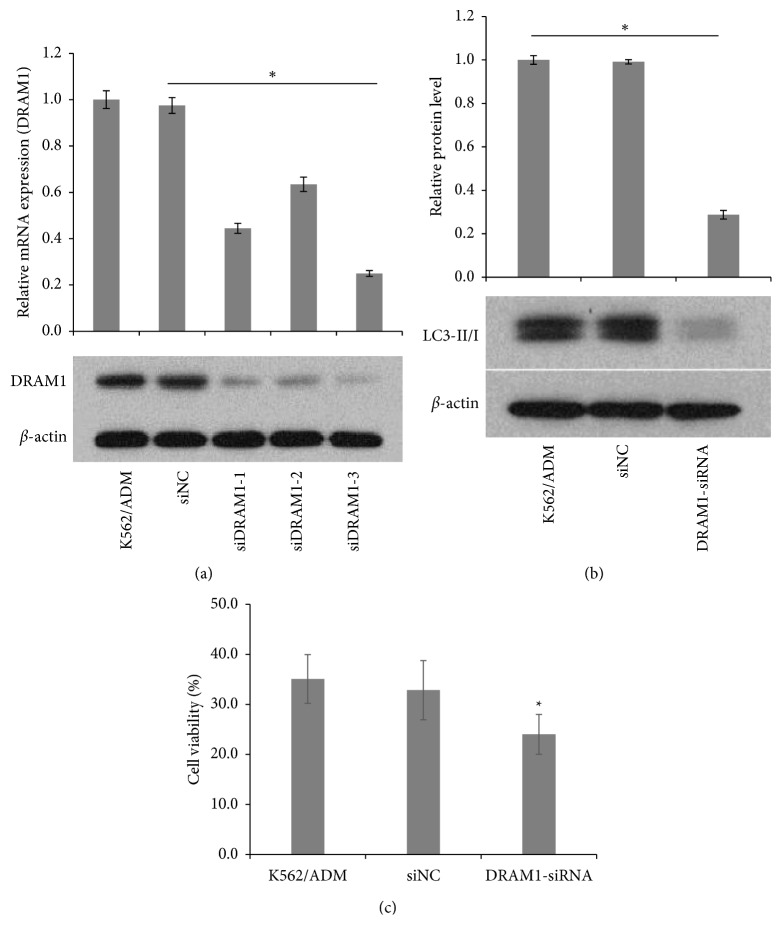
(a) K562/ADM cells were transiently transfected with three DRAM1 siRNAs or the siNC. Downregulation of DRAM1 was confirmed by both qPCR and western blot (*n* = 3; ^*∗*^*P* < 0.05). (b) Western blot was used to examine LC3II/I expression after K562/ADM cells were transfected with DRAM1-siRNA (*n* = 3; ^*∗*^*P* < 0.05). (c) CCK-8 assay was used to examine the cell viability after K562/ADM cells were transfected with DRAM1-siRNA and subsequently treated with 140 *μ*g/ml of ADM for 48 h (*n* = 3; ^*∗*^*P* < 0.05).

DRAM1 has been reported to play an essential role in TP53-dependent autophagy activation [36]. However, in the present study, to determine whether DRAM1 influenced leukemia cells autophagy and ADM resistance, K562/ADM cells expressing a relatively higher level of DRAM1 were selected for siRNA-mediated knockdown. We constructed three DRAM1 siRNA expression vectors and transfected K562/ADM cells with them. The interference sequence of DRAM1 siRNAs is listed in Table 3. As shown in Figure 9(a), the third siRNA of DRAM1 (DRAM1-homo-933), which exhibited the best inhibition effect both in mRNA and protein level, was chosen in the following studies. Western blot results showed that DRAM1 silence significantly decreased the expression of LC3II/I ratio (Figure 9(b)). Moreover, the CCK-8 cell proliferation assay indicated that K562/ADM cell viability was remarkably reduced by 15–20% upon ADM treatment compared with the siNC group, indicating DRAM1 suppression exerted important functions in improving drug resistance in leukemia cells (Figure 9(c)). Collectively, these data demonstrated that DRAM1 silencing downregulated the cell autophagy activity and reduced drug resistance to ADM in K562/ADM cells, indicating DRAM1 is a functional target gene of miR-199a-5p to regulate chemoresistance in AML cells.

## 4. Discussion

In the present study, we demonstrated that miR-199a-5p is markedly downregulated in refractory/relapsed AML patients compared with those who achieved complete remission following chemotherapeutic treatment. Consistent with the clinical data, we also found a significantly lower level of miR-199a-5p expression in ADM-resistant AML cell lines than ADM-sensitive cells. Enforced expression of miR-199a-5p restored the cells chemosensitivity to ADM. Moreover, we investigated that miR-199a-5p expression is inversely correlated with the autophagy level which is presented as a protective factor to the proliferation of leukemia cells on chemotherapeutic drugs. Meanwhile, we identified a potential regulatory correlation between miR-199a-5p and autophagy which is partially due to inhibition of the DRAM1 gene acting as a functional target of miR-199a-5p to modulate chemoresistance in AML cells.

Previous studies have reported that miR-199a-5p, functioning as a cancer suppressor, is downregulated in multiple malignant solid tumors such as hepatocarcinoma [7], colorectal cancer [10], breast cancer [37], ovarian cancer [8], osteosarcoma [9], bladder urothelial carcinoma [38], papillary thyroid carcinoma [39], and prostate adenocarcinoma [40]. Accumulating evidence has linked aberrant miR-199a-5p expression to acquired chemoresistance in some cancer types [9, 14, 15, 17]. For instance, silencing of miR-199a-5p was found to be involved in chemoresistance in ovarian cancer by regulating IKKB expression [41]. Overexpression of miR-199a-5p in hepatocellular carcinoma was shown to modulate tumor cells sensitivity to doxorubicin by targeting the MET-oncogene [7]. Abnormally upregulated in CTX-resistant colon carcinoma cells, miR-199a-5p is also reported to affect cancer cell resistance by targeting PHLPP1 [42]. Although miR-199a-5p may regulate chemoresistance by targeting different genes in different cancer types, the mechanisms could be generally summarized as cell cycle arrest, proliferation decrease, invasion prevention, and apoptosis inhibition [7, 8, 35, 41–43]. However, several recent research studies indicated that miR-199a-5p is implicated in chemoresistance of cancer cells via inhibition of autophagic pathways [9, 14]. In this regard, autophagy is activated as a protective mechanism to mediate the acquired resistance phenotype of some cancer cells during chemotherapy. Thus, the inhibition of autophagy can resensitize previously resistant cancer cells and increase therapeutic efficacy of chemotherapy. However, data for miR-199a-5p expression and function, especially the correlation between miR-199a-5p and autophagy, in leukemia cells are very limited.

It is reported that miR-199a/b-5p enhances imatinib efficacy via repressing WNT2 signaling-mediated protective autophagy in imatinib-resistant chronic myeloid leukemia cells [44]. Very recently, Aranda et al. also reported that miR-199a-5p overexpression inhibited the lysosome acidification and reduced autophagosomal degradation, which are a part of the mechanisms for miR-199a-5p to regulate retrograde transport and endolysosomal system in mammalian cells. Their results indicated miR-199a-5p as a negative regulator of autophagy [45]. Yet, the roles of both miR-199a-5p and autophagy in drug-resistant mechanisms of acute myeloid leukemia cells are still unclear. In our present study, we use human erythroleukemia cells K562 and their ADM-resistant counterparts K562/ADM, as the acute myeloid leukemia cell model to explore the expression of miR-199a-5p and autophagy and their interaction. We found that miR-199a-5p was downregulated, accompanied by an elevated autophagy level, in K562/ADM cells compared to parent cells. Consistent with the reported results by Wang et al. [46], we identified that the basic autophagy level and the ADM-induced autophagic activity in K562/ADM cells was higher than that in K562 cells, indicating that K562/ADM cells possess potent resistance to extra stressors. Furthermore, administration of miR-199a-5p mimics in K562/ADM cells markedly suppressed the expression of autophagy-related genes and sensitized the cells to ADM-induced cytotoxicity. Conversely, transfection of the miR-199a-5p inhibitor into K562 cells elicited the reverse effect, that is, enhanced autophagy levels and promoted ADM resistance. Particularly, autophagic flux analysis exhibited that miR-199a-5p negatively regulates autophagy through preventing autophagy initiation rather than blocking autophagosome degradation. Taken together, the above results revealed an inverse regulatory correlation between miR-199a-5p and autophagy both of which are shown to play important roles in acquired chemoresistance of AML cells.

Most miRNAs carry out their biological function by binding to their target molecules [47]. To investigate how miR-199a-5p affects AML cells autophagy, bioinformatic tools were used to search for potential targets of miR-199a-5p. Among the targets of miR-199a-5p, the damage-regulated autophagy modulator 1 (DRAM-1) belongs to an evolutionarily conserved family of lysosomal proteins, encodes a series of p53-inducible splice variants, and positively regulates autophagy in a p53-dependent manner [48]. It has been reported that DRAM1-mediated autophagic apoptosis is inhibited by activation of the PI3K/AKT signaling pathway in hepatoma [49], which also plays a crucial role in drug-resistant AML. Remarkably, Kerley-Hamilton et al. presented that DRAM1 expression is upregulated in response to cisplatin treatment in breast cancer cells [50]. Another study, in accordance with the above results, proposed that the upregulated DRAM1 expression may have significance in determining the sensitivity of breast cancer cells to cisplatin treatment [51]. It is also previously reported that DRAM1 might be suppressed by miR-199a-5p as a target gene to protect hepatocytes from ER stress-induced apoptosis according to DAVID database [52]. In the current study, we found DRAM1 mRNA and protein expression were decreased by transfection of miR-199a-5p mimics in K562/ADM cells and increased by miR-199a-5p inhibitors in K562 cells, suggesting that DRAM1 expression could be regulated by miR-199a-5p through both transcriptional and posttranscriptional inhibition. In addition, we further identified DRAM1 as a direct target of miR-199a-5p of K562/ADM cells through the luciferase reporter assay showing miR-199a-5p suppressed DRAM1 expression by binding to DRAM1 3′UTR directly. More notably, our findings demonstrated that DRAM1 is a functional target of miR-199a-5p in regulating autophagy and chemoresistance because downregulation of the DRAM1 gene by siRNA resulted in autophagy suppression and chemosensitivity restoration in K562/ADM cells. Yi et al. also reported DRAM1 as a target gene of miR-199a-5p in human breast cancer cells. They showed that miR-199a-5p was closely involved in basal and irradiation-induced autophagy through direct action of 3′UTR of DRAM1 gene and could affect radiation sensitivity of breast cancer. Interestingly, they found that miR-199a-5p behaved completely opposite with regards to trends in two different human breast cancer cell lines. Their findings indicated the impact of miR-199a-5p on DRAM1 gene might be complicated and cell line specific [17]. However, there may be other molecules which are also targeted by miR-199a-5p in the gene regulatory network of acquired drug resistance in leukemia and other tumor cells. Previous studies demonstrated ATG7 and mTOR as the target genes of miR-199a-5p involved in the regulation of the autophagic process in hepatocellular carcinoma and hypertrophic hearts [14, 16]. These molecular targets may work through the mechanisms of modulation in different autophagic stages and interconnection between autophagy and apoptosis.

In conclusion, the current study described for the first time that miR-199a-5p negatively regulates the autophagy level, both of which are implicated in acquired chemoresistance in AML. Moreover, DRAM1 is a novel functional target gene, through which miR-199a-5p could probably, at least in part, modulate autophagy. Therefore, targeting the miR-199a-5p/DRAM1/autophagy axis might be a valuable therapeutic strategy in ADM-resistant AML patients.

## Figures and Tables

**Table 1:** Characteristics of AML patients.

	RR (*n* = 32)	CR (*n* = 11)	*P*
Sex (male/female)	17/15	6/5	0.126
Median age (range)	42 (23–54)	49 (28–58)	0.481
FAB classification			
M1	3	1	
M2	8	3	
M3	0	0	
M4	7	2	
M5	12	5	
M6	2	0	

RR = relapsed/refractory; CR = complete remission.

**Table 2:** Primers for miR-199a-5p, DRAM1, and reference genes.

Gene	Primer	Sequence (5′ ⟶ 3′)
miR-199a-5p	Forward	GCCAAGCCCAGTGTTCAGAC
	Reverse	GTGCAGGGTCCGAGGTATTC
U6	Forward	CTCGCTTCGGCAGCACA
	Reverse	AACGCTTCACGAATTTGCGT
DRAM1	Forward	GATTGGTGGGATGTTTCGG
	Reverse	GAGATGATGGACTGTAGGAGC
*β*-actin	Forward	CTTAGTTGCGTTACACCCTTTCTTG
	Reverse	CTGTCACCTTCACCGTTCCAGTTT

**Table 3:** Interference sequence of DRAM1 siRNAs.

Name	Sequence (5′ ⟶ 3′)	Length (bp)
DRAM1-homo-579	CUCC CGUA UAUC AGUG AUAT T	21
DRAM1-homo-666	GCCA CGAU GUAU ACAA GAUT T	21
DRAM1-homo-933	GCCA CAUA CGGA UGGU CAUT T	21

## Data Availability

All original data supporting the conclusions of this study are available from the corresponding author upon request. All the figures are based on the statistical analyses of the original data.
